# Evaluation of a Patient-Centered Fall-Prevention Tool Kit to Reduce Falls and Injuries: A Nonrandomized Controlled Trial

**DOI:** 10.1001/jamanetworkopen.2020.25889

**Published:** 2020-11-17

**Authors:** Patricia C. Dykes, Zoe Burns, Jason Adelman, James Benneyan, Michael Bogaisky, Eileen Carter, Awatef Ergai, Mary Ellen Lindros, Stuart R. Lipsitz, Maureen Scanlan, Shimon Shaykevich, David Westfall Bates

**Affiliations:** 1 Center for Patient Safety, Research and Practice, Division of General and Internal Medicine and Primary Care, Brigham and Women’s Hospital, Boston, Massachusetts; 2 Harvard Medical School, Harvard University, Boston, Massachusetts; 3 School of Nursing, Columbia University, New York, New York; 4 Columbia University Irving Medical Center/New York–Presbyterian, New York, New York; 5 Institute of Healthcare Systems Engineering, Boston, Massachusetts; 6 Montefiore Medical Center Hospitals, Bronx, New York; 7 Kennesaw State University, Kennesaw, Georgia

## Abstract

**Importance:**

Falls represent a leading cause of preventable injury in hospitals and a frequently reported serious adverse event. Hospitalization is associated with an increased risk for falls and serious injuries including hip fractures, subdural hematomas, or even death. Multifactorial strategies have been shown to reduce falls in acute care hospitals, but evidence for fall-related injury prevention in hospitals is lacking.

**Objective:**

To assess whether a fall-prevention tool kit that engages patients and families in the fall-prevention process throughout hospitalization is associated with reduced falls and injurious falls.

**Design, Setting, and Participants:**

This nonrandomized controlled trial using stepped wedge design was conducted between November 1, 2015, and October 31, 2018, in 14 medical units within 3 academic medical centers in Boston and New York City. All adult inpatients hospitalized in participating units were included in the analysis.

**Interventions:**

A nurse-led fall-prevention tool kit linking evidence-based preventive interventions to patient-specific fall risk factors and designed to integrate continuous patient and family engagement in the fall-prevention process.

**Main Outcomes and Measures:**

The primary outcome was the rate of patient falls per 1000 patient-days in targeted units during the study period. The secondary outcome was the rate of falls with injury per 1000 patient-days.

**Results:**

During the interrupted time series, 37 231 patients were evaluated, including 17 948 before the intervention (mean [SD] age, 60.56 [18.30] years; 9723 [54.17%] women) and 19 283 after the intervention (mean [SD] age, 60.92 [18.10] years; 10 325 [53.54%] women). There was an overall adjusted 15% reduction in falls after implementation of the fall-prevention tool kit compared with before implementation (2.92 vs 2.49 falls per 1000 patient-days [95% CI, 2.06-3.00 falls per 1000 patient-days]; adjusted rate ratio 0.85; 95% CI, 0.75-0.96; *P* = .01) and an adjusted 34% reduction in injurious falls (0.73 vs 0.48 injurious falls per 1000 patient-days [95% CI, 0.34-0.70 injurious falls per 1000 patient-days]; adjusted rate ratio, 0.66; 95% CI, 0.53-0.88; *P* = .003).

**Conclusions and Relevance:**

In this nonrandomized controlled trial, implementation of a fall-prevention tool kit was associated with a significant reduction in falls and related injuries. A patient–care team partnership appears to be beneficial for prevention of falls and fall-related injuries.

**Trial Registration:**

ClinicalTrials.gov Identifier: NCT02969343

## Key Points

**Question:**

Is a fall-prevention tool kit that engages patients and families associated with a reduction in falls?

**Findings:**

In this nonrandomized controlled trial including 37 231 patients from 14 medical units within 3 academic medical centers, an interrupted time series found that implementation of a fall-prevention tool kit was associated with a statistically significant 15% reduction in overall inpatient falls and a 34% reduction in injurious falls.

**Meaning:**

The findings suggest that tools to support patient engagement throughout hospitalization in the fall-prevention process may be associated with a reduction in falls and fall-related injuries.

## Teaser

This nonrandomized controlled trial assesses whether implementation of a nurse-led, patient-centered tool kit for fall prevention was associated with a reduction in rates of falls and fall-related injuries in 3 academic medical centers in Boston and New York City.

## Introduction

Falls represent a leading cause of preventable injury.^1^ Hospitalized patients are at an increased risk for falls, which may result in serious injuries, such as hip fractures, subdural hematomas, or even death.^2,3^ Injurious falls are associated with increased hospital stays of 6 to 12 days,^4^ and the costs of serious episodes of injury range from $19 376 to $32 215 (2019 USD).^5^ Patient falls and related injuries are considered nursing-sensitive indicators because fall prevention depends on the quantity and quality of nursing care.^6,7,8^ Most falls in hospitals are preventable,^9^ and resultant injuries are not reimbursed by the Centers for Medicare & Medicaid Services.^10^ Multifactorial strategies can reduce rates of falls in hospitals, although the evidence for reducing fall-related injuries is inconclusive owing to the limited number of clinical trials that have assessed this outcome.^11^ To our knowledge, no prior multisite evaluation in acute care hospitals has shown a significant reduction in injurious falls.

A previous study^12^ theorized that fall prevention in hospitals was a 3-step process: (1) assessing fall risk, (2) developing a personalized prevention plan, and (3) executing the plan consistently. Our team developed the Fall Tailoring Interventions for Patient Safety (TIPS) tool kit, a nurse-led, evidence-based fall-prevention intervention that uses bedside tools to communicate patient-specific risk factors for falls and a tailored prevention plan. The tool kit provides care team members with the information they need to routinely engage in the fall-prevention process.^12^ In a randomized clinical trial within a single health care system, Fall TIPS reduced patient falls by 25%, but there was no difference noted in fall-related injuries.^13^ A follow-up case-control study suggested that falls within the intervention units were largely attributable to patients’ nonadherence to their fall-prevention plan^14^ and that further strategies are needed for engaging patients in the 3-step fall-prevention process during hospitalization.

**Figure 1.**
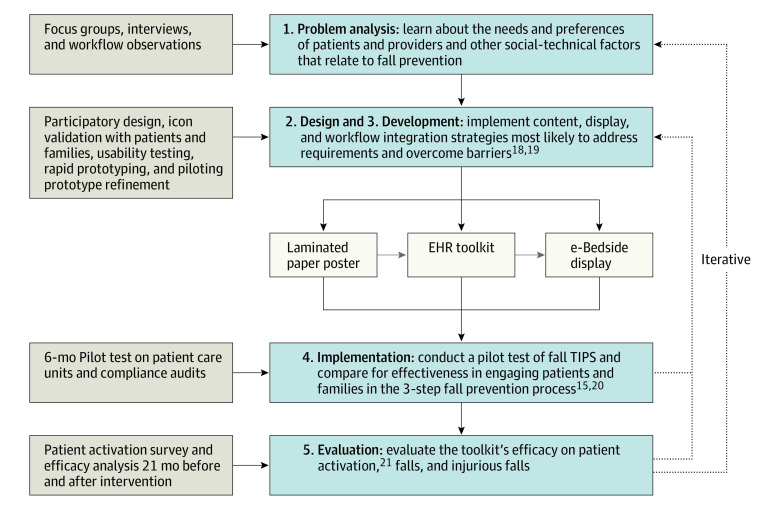
Five-Phase Intervention Development and Evaluation Unit staff and patients were engaged in developing, refining, implementing, and pilot testing a patient-centered Fall Tailoring Interventions for Patient Safety (TIPS) tool kit with high-tech and low-tech modalities. EHR indicates electronic health record.

In collaboration with Northeastern University’s Healthcare Systems Engineering Institute, we conducted observational and qualitative research with hospitalized inpatients, family members, and health care professional to make the Fall TIPS tool kit more patient-centered and to address barriers to engaging patients and families in the 3-step fall-prevention process.^15,16^ The project was divided into the 5 following iterative phases using the Reach, Effectiveness, Adoption, Implementation, and Maintenance (RE-AIM) framework^17^ (Figure 1): (1) problem analysis using workflow observations and individual and group interviews^18^; (2) design using knowledge gained in phase 1 to plan a patient-centered Fall TIPS tool kit with multiple modalities^18,19^; (3) development using participatory design, rapid prototyping, computer modeling, and simulation methods to construct the patient-centered Fall TIPS tool kit^18,19^; (4) implementation and pilot testing of the tool kit in patient care units^19,20^; and (5) evaluation of the association of the tool kit with patient activation.^21^ The end result was a tool kit that included high-tech and low-tech Fall TIPS modalities, can be used by nursing staff and integrated into various hospital workflows, and supports patient activation and engagement in the 3-step fall-prevention process.^20,21^ Modalities included (1) a laminated paper poster,^19^ (2) a tool kit integrated with the electronic health record (EHR),^13^ and (3) an electronic bedside screen (e-bedside) display.^20^ From September 2014 to September 2015, unit staff were involved in developing, refining, and piloting the intervention, testing its association with patient activation in the fall-prevention plan (phases 1-5 above and Figure 1) and selecting the modality they would implement. At the end of this period, the laminated paper poster and the refined EHR-integrated tool kit modalities were complete. The e-bedside display design was complete, but this modality required additional EHR integration and was not available for implementation until October 1, 2016. Nine units chose to implement the laminated paper poster, 2 chose the EHR-integrated tool kit, and 3 chose the e-bedside display modality. The goal of the trial was to assess whether a fall-prevention tool kit that engages patients and families in the fall-prevention process throughout hospitalization is associated with reduced falls and injurious falls.

## Methods

### Overall Design

**Figure 2.**
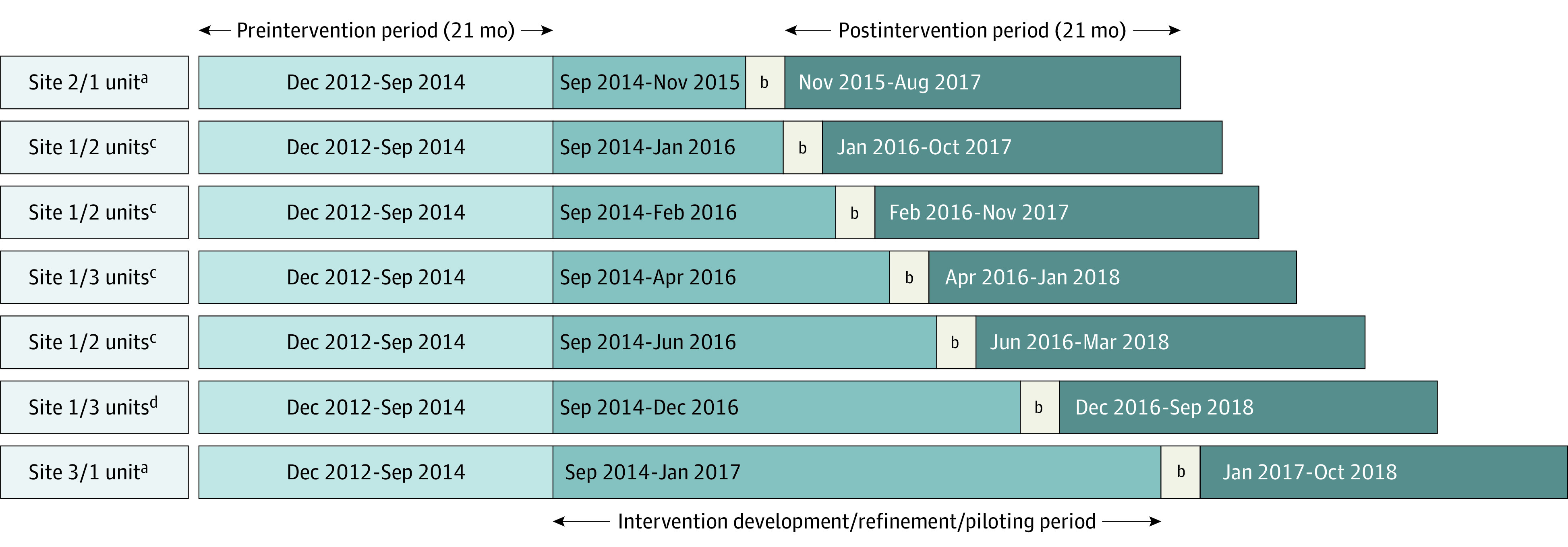
Nonrandomized Stepped-Wedge Design for Fall Tailoring Interventions for Patient Safety (TIPS) Implementation by Modality Problem analysis, design, development, pilot implementation, and evaluation periods were inserted into the interrupted time-series analysis to account for potential confounders associated with developing the intervention. Start dates were assigned to each unit based on the selected Fall TIPS modality and unit-based constraints. Regardless of start date, each unit contributed 21 weeks of preintervention data and was followed up for 21 weeks after a 2-month implementation and wash-in period. ^a^Electronic health record. ^b^Two-month implementation and wash-in period. ^c^Laminated paper poster. ^d^Electronic bedside display.

This nonrandomized controlled trial (NCT02969343) used a stepped-wedge design (Figure 2). The trial protocol is given in Supplement 1. Owing to active staff engagement in the problem analysis, design, development, pilot implementation, and evaluation phases (Figure 1), data from these phases were not included in the analysis. Each unit served as its own control. Randomization of unit start dates was not done for practical reasons, including constraints in unit operations owing to pending go-live dates of new EHR systems at all 3 hospitals and other concurrent projects. The research team assigned start dates to each unit based on the Fall TIPS modality selected, and these constraints (ie, EHR modalities) were tied to EHR go-live dates. Regardless of start date, each unit contributed 21 weeks of preintervention data and was followed up for 21 weeks after a 2-month implementation and wash-in period (Figure 2). The study was approved by the Partners HealthCare Human Subjects Committee of Brigham and Women’s Hospital, the Human Research Protection Office of Columbia University, and the Montefiore Einstein Office of Clinical Trials. Owing to the quality-improvement nature of the intervention, a waiver of informed consent was granted by the institutional review boards of Brigham and Women’s Hospital, New York–Presbyterian, and Montefiore Medical Center. The study followed the Transparent Reporting of Evaluations With Nonrandomized Designs (TREND) reporting guideline.^22^

### Unit Selection and Participants

An interrupted time-series evaluation of the patient-centered Fall TIPS tool kit was conducted among 37 231 patients in 14 adult medical units in 3 academic medical centers: site 1 (Boston, Massachusetts), site 2 (Bronx, New York), and site 3 (New York, New York) between November 1, 2015, and October 31, 2018. The purpose was to evaluate the tool kit’s effectiveness and compare the rates of falls and falls with injury from a 21-month preintervention period and a 21-month postintervention period (Figure 2). Site 1 agreed to implement Fall TIPS in all 12 medical units. Sites 2 and 3 each agreed to implement Fall TIPS in 1 acute care medical unit with rates of falls and injuries that were above average for their institutions.

### Study Design and Intervention

In collaboration with unit leadership, the study team assigned the month when the intervention would go live between September 2015 and November 2016 based on the modality selected and associated constraints (Figure 2). Previous testing revealed that all modalities were effective in facilitating patient engagement in the 3-step fall-prevention process.^20^ An 11-by-17-inch laminated Fall TIPS poster was displayed at the bedside and used color-coded clinical decision support to link the Morse Fall Scale^9^ risk factors to evidence-based interventions. Nurses completed the poster with a dry-erase marker at admission and during each shift with the patient and family (if available) and posted it at the bedside. Using the Fall TIPS EHR-integrated tool kit, nurses identified patient-specific risk factors using the Morse Fall Scale,^9^ and clinical decision support automatically linked each risk factor with the appropriate preventive interventions. Nurses could further tailor prevention plans based on their knowledge of the patient. Once completed, posters (8.5 × 11 in) detailing the risk factors and fall-prevention plan were generated and printed from the EHR system, hung at the bedside (sites 2 and 3), or automatically displayed on the bedside computer screensaver (e-bedside display, site 1) and reviewed with the patient and family at admission and during each shift.

Methods for stakeholder engagement and implementation in study units are described elsewhere.^19^ In brief, study staff engaged leadership at institutional and care-unit levels through presentations on the evidence supporting Fall TIPS. We used a peer-champion model of existing unit-based nursing staff for education and training.^19^ Nurse champions who completed competency training were involved in continuous engagement of staff nurses, monitoring of fidelity, and reinforcement, with the intention of successful integration of the intervention into practice.^19^ Study staff visited study units to provide training during the go-live week.^19^ Unit-based nurse champions measured adherence to the protocol with patient engagement audits consisting of 3 questions: (1) Is the Fall TIPS poster updated with the correct patient information? (2) Can the patient/family express their fall risk factors? and (3) Can the patient/family express their fall-prevention plan? Based on continuous feedback from unit champions, barriers to adoption and spread were addressed.^19^ After the go-live date, nurse champions completed 5 random audits per month and provided peer feedback to the nurses caring for the audited patients.

### Outcomes

The primary outcome measure was the overall rate of patient falls per 1000 patient-days during the study period. The overall rate of falls with injury per 1000 patient-days was the secondary outcome. Data on falls and resulting injury levels are routinely recorded in an event reporting system at all participating hospitals and were used in the analysis.

### Statistical Analysis

The association between the intervention and the rate of patient falls and falls with injury per 1000 patient-days on the unit was analyzed using Poisson regression (for rates) estimated with overdispersion via generalized estimating equations to account for clustering within a unit using an exchangeable correlation for patients within the same unit. In the Poisson regression models, we fit segmented lines for the 2 periods (before and after intervention) to test for the statistical significance of observed changes in the fall rates in the interrupted time series associated with the intervention. In the Poisson regression model for rates with clustering by unit, we adjusted for the following patient-level characteristics: sex (as classified in the EHR), race/ethnicity, insurance (public or private), age at admission, and binary Charlson Comorbidity Index score (0-1 or ≥2). For the Poisson regression parameters to be interpreted as log rate ratios, unit length of stay was used as an offset term with Poisson modeling.

In a secondary analysis to assess whether the changes before vs after intervention differed by age group (younger than 65 years vs 65 years or older), we fit the adjusted Poisson regression model for rates with an interaction between age group and period. In another secondary analysis to assess whether the changes from before the intervention to after the intervention differed by site, we fit the adjusted Poisson regression model for rates with an interaction between site and period.

Patient characteristics in the 2 periods are presented as means for continuous variables and proportions for categorical variables. Balance in patient characteristics in the 2 periods was assessed using standardized differences. All analyses used the intention-to-treat principle. Statistical significance was set at *P* < .05 using a 2-sided test. We used SAS statistical software, version 9.4 (SAS Institute), for the analyses.^23,24^

## Results

The study included 37 231 patients and 277 655 patient-days; 17 948 patients were included in the preintervention period and 19 283 in the postintervention period (Table). Patients in both periods were similar regarding age, sex, race/ethnicity, primary insurance type, hospital and unit length of stay, and Charlson Comorbidity Index score at admission. A total of 9723 (54.17%) patients during the preintervention period and 10 325 (53.54%) during the postintervention period were women, and 9760 (62.57%) patients during the preintervention and 10 521 (60.17%) during the postintervention period were White. The mean (SD) age of patients was 60.56 (18.30) years in the preintervention period and 60.92 (18.10) years in the postintervention period. The mean (SD) hospital length of stay was 7.53 (9.04) days in the preintervention period and 7.39 (10.03) days in the postintervention period. All standardized differences comparing demographics across periods were less than 10% (Table), suggesting that the demographics were well balanced over periods.^23,24^ Nevertheless, to protect against possible confounding, we adjusted for all demographics in the interrupted time-series analyses. There were no statistically significant trends from month to month within the preintervention or postintervention periods in relation to falls or falls with injury. Therefore, we compared adjusted rates across the preintervention and postintervention periods. After Fall TIPS implementation, site 1 had a mean compliance rate of 86% on the 3-question audit, and sites 2 and 3 had mean compliance rates greater than 95%. This translated into a clinically significant patient-centered Fall TIPS intervention in all study units.^20^

**Table.** Patient Characteristics and Standardized Differences Before and After Implementation of the Fall TIPS Tool Kit Intervention

Characteristics	Before the intervention, No.	After the intervention, No.	Standardized difference (%)^a^
Patient-days, No.	135 163	142 492	NA
Patients, No.	17 948	19 283	NA
Hospital length of stay, mean (SD)	7.53 (9.04)	7.39 (10.03)	1.47
Unit length of stay, mean (SD)	5.86 (6.07)	5.88 (7.45)	–0.29
Age, mean (SD)	60.56 (18.30)	60.92 (18.10)	–1.98
Women, No. (%)	9723 (54.17)	10 325 (53.54)	1.26
Race/ethnicity, No. (%)			
White	9760 (62.57)	10 521 (60.17)	4.93
Other^b^	5843 (37.46)	6971 (39.87)	–4.93
Missing	2349	1797	NA
Primary insurance, No. (%)			
Public	12 455 (70.84)	12 754 (70.14)	1.53
Private	5126 (29.16)	5429 (29.86)	–1.53
Missing	285	1797	NA
Total Charlson Comorbidity Index score at admission, No. (%)			
0-1	8039 (44.79)	7953 (41.25)	7.15
≥2	9909 (55.21)	11 328 (58.75)	–7.15
Missing	0	2	NA

Abbreviatons: NA, not applicable; TIPS, Tailoring Interventions for Patient Safety.

^a^ Standardized differences with absolute values of less than 10% reflect well-balanced covariates across periods.^23^

^b^ Other included Black, Asian, and Native American.

In the adjusted analysis, the overall fall rate in study units decreased from 2.92 falls per 1000 patient-days (95% CI, 2.53-3.36 falls per 1000 patient-days) before implementation to 2.49 falls per 1000 patient-days (95% CI, 2.06-3.0 falls per 1000 patient-days) in the postintervention period. After adjustment for demographics in the Poisson regression model, study units using the patient-centered Fall TIPS tool kit achieved a 15% reduction in patient falls in the postintervention period (adjusted rate ratio [RR], 0.85; 95% CI, 0.75-0.96; *P* = .01). In the subanalysis by age, the decrease in falls was largest for patients younger than 65 years; units achieved an 18% reduction in patient falls in this age group in the postintervention period (adjusted RR, 0.82; 95% CI, 0.70-0.97; *P* = .02) vs a 10% reduction for patients age 65 and older (adjusted RR, 0.90; 95% CI, 0.74-1.09; *P* = .28), with the latter difference not being statistically significant.

**Figure 3.**
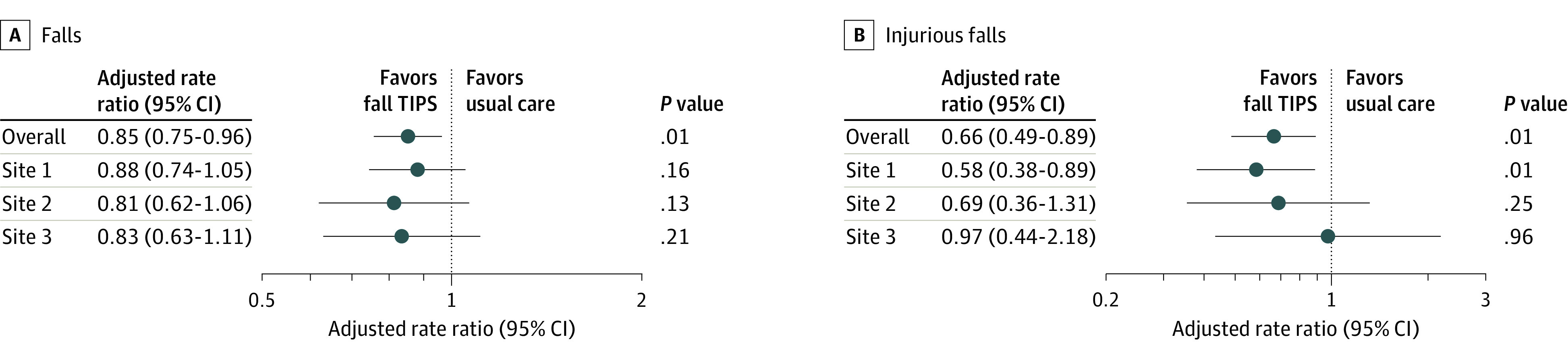
Adjusted Rate Ratios of Falls and Injurious Falls by Site Before vs After Fall Tailoring Interventions for Patient Safety (TIPS) Intervention The adjusted rate ratios were obtained from a Poisson regression model with overdispersion and clustering by unit, adjusted for the following patient-level characteristics: sex, race/ethnicity, insurance (public vs private), age at admission, and binary Charlson comorbidity score (0-1; ≥2). Unit length-of-stay was used as an offset term with Poisson modeling so rates could be interpreted as events per patient length of stay.

In the adjusted analysis, the overall injurious fall rate in study units decreased from 0.73 injurious falls per 1000 patient-days (95% CI, 0.59-0.92 falls per 1000 patient-days) before implementation to 0.48 injurious falls per 1000 patient-days (95% CI, 0.34-0.70 falls per 1000 patient-days) in the postintervention period. After adjustment for demographics in the Poisson regression model, study units achieved a 34% reduction in overall falls with injury in the postintervention period (adjusted RR, 0.66; 95% CI, 0.53-0.88; *P* = .003). The rate ratios for falls and injurious falls before and after the intervention are shown in Figure 3. In the subanalysis by age, the decrease in injurious falls was largest for patients aged 65 years or older, among whom units achieved a 48% reduction in the postintervention period (adjusted RR, 0.52; 95% CI, 0.34-0.82; *P* = .004) vs a 19% reduction for patients younger than 65 (adjusted RR, 0.81; 95% CI, 0.54-1.19; *P* = .28), with the latter difference not being statistically significant.

## Discussion

We evaluated a nurse-led intervention focused on engaging patients and families with the care team at 3 institutions and found that the intervention was associated with overall reduced rates of falls and fall-related injuries. Previous quality improvement studies^25,26,27^ have shown a reduction in injuries but not in acute-care units in multiple geographic locations. This study suggests that hospital-based fall-prevention interventions are associated with reduced rates of falls when they routinely engage patients and families in the fall-prevention plan.

These findings build on research supporting patient engagement in safety initiatives, which has been associated with improved quality, safety, patient experience, and empowerment.^28,29^ Patients are prepared to carry out specific and actionable interventions recommended by health care professionals when they are engaged in the process.^30,31^ As shown in previous work,^20,21^ both high-tech and low-tech tools can facilitate patient engagement in the fall-prevention plan. Patient engagement in the 3-step fall-prevention process results in a partnership between the patient and care team and strengthens the Fall TIPS tool kit^13^ intervention.

In the subanalysis, we found that the intervention was associated with reduced falls in younger patients and with reduced fall-related injuries in older patients. These results differ from another evaluation,^13^ in which the tool kit was associated with reduced falls in older patients and there was no difference in the injurious fall rate. Interviews with younger patients revealed that they did not believe that they were at risk for falls in the hospital, especially those who were independent at home.^32^ We refined the tool kit to improve patient engagement in the 3-step fall-prevention process. Our rationale was that if patients were included in risk assessment and the development of their prevention plan, they would be more likely to believe that they are at risk for falls in the hospital and perhaps more likely to follow their prevention plan. The findings suggest that engaging patients in the fall-prevention process is important because this simple practice was associated with fewer falls among younger patients and substantially fewer fall-related injuries among older patients—those at greatest risk of injury.

### Strengths and Limitations

This study has strengths. Inclusion of 3 academic medical centers with many patients and different patient populations enhanced the generalizability of the Fall TIPS tool kit. Engagement of leadership at both the institutional and care-unit levels was important for the integration of the intervention into practice. Fidelity was high owing to unit champions and staff nurse engagement through continuous monitoring and peer feedback. Unit-based nurse champions had a key role in discovering and addressing barriers to use of the tool kit, which proved to be vital to the success and sustainability of the intervention.

This study also has limitations. There are challenges to conducting pragmatic studies that engage stakeholders in intervention development in complex clinical settings. Despite evidence that participatory design and development with end users strengthen interventions, they also make quantifying the association between the intervention and a reduction in falls more difficult.^33^ Methods in the early phases of this project included extensive clinician and patient involvement in developing, refining, and pilot testing the patient-centered Fall TIPS tool kit (Figure 1). Iteratively changing processes could have impacted practice and outcomes. To account for this, we evaluated the intervention using an interrupted time series design and removed the problem analysis, design, development, and pilot implementation phases that began before the first prototypes of the Fall TIPS tool kit were developed and extended until the Fall TIPS tool kit modalities’ design was complete. We included a wash-in period 2 months after going live in each study unit. This was the time it took nurses on clinical units to fully integrate the tool kit and consistently submit compliance audits.

We assessed the effectiveness of the patient-centered Fall TIPS tool kit within existing institutional infrastructures and workflows. One limitation is that support from hospital leadership and unit champions, communication channels, timing of implementation, and nurse and patient adherence to the protocol were variables that could not be fully controlled. We had originally planned to randomize the go-live dates for site 2 (Supplement 1), but the decision to implement a new EHR at each site after the start of the study and the decision to allow clinicians to select the Fall TIPS modality that best fit unit workflow limited the ability to randomize. Although the study design did not allow for perfect comparability, it revealed valuable information about the generalizability of the tool kit and its effectiveness in diverse, real-world acute care environments for a relatively long duration (21 months). Although the multisite evaluation is a strength of the study, limiting the evaluation to a single unit at sites 2 and 3 is a limitation. A larger evaluation is needed to fully evaluate generalizability. We acknowledge that there are overlapping 95% CIs in the secondary analyses by site and age. However, examining the overlap between 95% CIs is a conservative approach to testing whether 2 groups are significantly different (compared with the *P* value for testing for differences in 2 groups). Others have shown that if the two 95% CIs overlap, it does not mean that the 2 groups are not significantly different.^34,35^

## Conclusions

In this nonrandomized controlled trial, implementation of a nurse-led, patient-centered fall-prevention tool kit was associated with reduced rates of falls and injurious falls. The fall-prevention tool kit helped link patient-specific risk factors to interventions most likely to prevent a fall.^20^ Various modalities of the tool kit allow for integration into existing clinical workflows in diverse hospital settings. This tool kit appears to addresses the gap among nursing assessment of fall risk, tailored fall-prevention interventions, and engagement of patients throughout the fall-prevention process.^13,36^

## Notes

Supplement 1. **Trial Protocol**

Supplement 2. **Data Sharing Statement**
