# Supplementary material for: Evaluation of a Patient-Centered Fall-Prevention Tool Kit to Reduce Falls and Injuries: A Nonrandomized Controlled Trial
Source: JAMA Netw Open. 2020 Nov 17;3(11):e2025889. doi: 10.1001/jamanetworkopen.2020.25889 (PMC7672520; doi:10.1001/jamanetworkopen.2020.25889)
Supplement: Supplement 1. — Trial Protocol [file jamanetwopen-e2025889-s001.pdf]

Building and Testing a Patient-Centric Fall Prevention Toolkit

ABSTRACT

Falls represent a leading cause of preventable injury in all healthcare settings and are a frequently reported serious adverse event. Falls are devastating to patients, family members, and providers. Most falls are preventable. In our previous work we have learned that preventing falls is a three step process: 1) identifying risk factors; 2) developing a tailored or personalized plan to decrease risk; and 3) consistently carrying out the plan. Our team designed a fall prevention toolkit that made it easy for professional and paraprofessional providers to consistently complete the three step fall prevention process. We found that our fall prevention toolkit reduced patient falls by 22%[1]. However, the literature suggests that 78% of falls are preventable[2, 3]. We hypothesize that to further reduce falls, we need to partner with patients and their family members so that the entire team can routinely participate in the prevention process. In this project, we propose to develop a patient-centered fall prevention toolkit that will actively engage patients and family in the three-step fall prevention process. Iterative participatory design methods will be used to involve patients and their family caregivers in developing a clear understanding of the problem, in design, and in development of a fall prevention toolkit. We will implement the toolkit within the existing Patient Centered Toolkit (PCTK) infrastructure at Brigham and Women's Hospital (BWH). After a pilot where we will evaluate usability of the toolkit in the context of acute care workflows, we will then use a prospective randomized cluster design to evaluate the effectiveness of the toolkit on medical and oncology units at BWH and using a pre/post pilot at Montefiore Medical Center in the Bronx with ethnically diverse patients. The RE-AIM (reach, effectiveness, adoption, implementation, and maintenance) framework[4, 5] will be used to inform the research questions and methods and to plan for implementation and dissemination from the time of project initiation. The outcomes of this project will be a patient-centered fall prevention toolkit and measurement of its impact on patients' perceptions of fall risk communication, care plan concordance, and patient falls/injurious fall trends. The fall prevention toolkit will be widely disseminated to prevent falls in hospitalized patients.

Specific Aims

1. To engage patients and their family caregivers in the design of a fall prevention toolkit.
    a. What components are needed to engage patients/family caregivers in the three steps of the fall prevention process?
2. To partner with patients, family and other stakeholders to implement and iteratively refine the toolkit for use during an acute hospitalization.
    a. What strategies are needed to successfully implement a patient-centered fall prevention toolkit?
3. To evaluate the effects of the intervention on patients' perceptions of fall risk communication, care plan concordance by patients and care team members, and on the incidence of patient falls and fall related injuries.

BACKGROUND AND SIGNIFICANCE

Falls are a leading cause of preventable injury in all healthcare settings and a frequently reported serious adverse event[6]. Hospitalized patients are at an increased risk for falls and these falls may result in injury and death[7, 8].An unfamiliar environment, acute illness, surgery, bed rest, medications, treatments, and the placement of various tubes and catheters are common factors that place patients at risk. A single fall may result in a fear of falling that can begin a downward spiral of reduced mobility, loss of function and additional risk for falls[9, 10]. Even falls without injury are costly (approximately $460 per patient[11]). Falls are devastating to patients, family members, and providers and are associated with many potential negative consequences [3, 9, 10, 12-14].

In addition to the physical and mental health costs of falls for the person and family, the productivity and economic costs of injurious falls are tremendous. In acute care settings, the annual costs related to falls are estimated at $1.08 billion and care provided for fall related injuries is not reimbursable by the Centers for Medicare and Medicaid Services (CMS) [11, 15]. This project is consistent with the CMS goal to reduce hospital acquired conditions by 40%, preventing 1.8 million injuries[12] and the Healthy People 2020 goal of preventing and reducing consequences of unintentional injuries[16].

Innovation and Preliminary Work

The proposed innovation builds on our previous work and directly addresses existing gaps[1, 17-19]. Approximately 78% of patient falls are preventable[2, 3]. Prior to our 2010 study[1], fall prevention in acute care hospitals had been studied for four decades and the results had been largely inconclusive[17, 20-22]. A

systematic review suggests that conducting a fall risk assessment and personalizing interventions based on the results of that assessment may prevent falls from occurring[17]. Our team conducted qualitative studies to explore the patient's and care provider's experience of a fall[23, 24]. We learned that patient falls are a consequence of suboptimal communication and that fall prevention is a 3-step process: 1) assessing risk, 2) developing a tailored or personalized plan, and 3) executing the plan consistently. Providing bedside tools to communicate patient-specific fall risk status and a personalized plan can ensure that all care team members have the information they need to routinely engage in the fall prevention process[24].

Based on these findings, our team developed a web-based Fall TIPS (Tailoring Interventions for Patient Safety) Toolkit (FTTK) and conducted testing in four Partners HealthCare (PHS) acute care hospitals. The FTTK uses a set of validated icons[25] (see **Figure 1**) to communicate fall risks/interventions in the form of personalized bed posters to replace the generic “high risk for falls” signs typically used in hospitals. In a randomized control trial involving over 10,000 patients, the FTTK reduced patient falls in acute care hospitals by 22% and was most effective with patients over age 64[1].

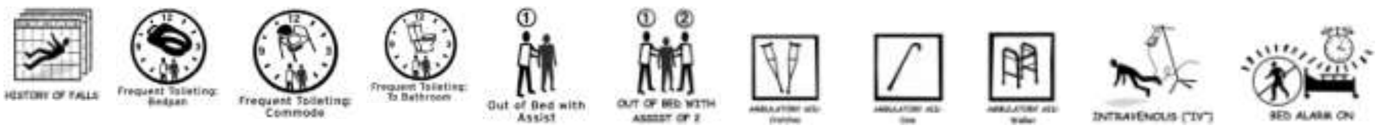

Figure 1: FTTK Icons

In a follow-up study[26] we looked at why some patients on intervention units fell, despite having access to the toolkit and found that the most frequent root cause was non adherence with the recommended plan by patients. For example, despite the FTTK recommendation for planned assistance, incident reports indicated that patients were frequently alone at the time of the fall. Other studies have also identified suboptimal patient adherence with fall prevention interventions. Nyberg reported that two-thirds of falls occurring on a rehabilitation unit related to lack of adherence with the fall prevention plan by care team members (9%) or more often, by patients (58%)[27]. During interviews with patients who had fallen in the hospital[23] we learned that patients and family caregivers want to be part of the team to prevent them from falling, but they are not consistently involved in developing and executing the fall prevention plan during a hospitalization[23, 24].

We hypothesize that by engaging patients and family caregivers via bedside tools we will see further reduction in the fall rate by virtue of improved adherence with the fall prevention plan. **The purpose of this study is to develop the tools, strategies, and techniques that are needed to engage patients and family caregivers in the 3-step fall prevention process: 1) assessing fall risks, 2) developing a personalized plan, and 3) executing that plan.** We will partner with the Systems Engineering, Usability, and Integration core to use rapid prototyping and other iterative methods that we used successfully to develop the FTTK for providers[28]. We will work with patients, family caregivers and other stakeholders to develop a fall prevention toolkit that they can use at the bedside to facilitate routine involvement in the fall prevention process. We will also leverage engineering expertise and tools to develop the best possible intervention. We will include all stakeholders to ensure that the toolkit will fit within existing workflows and overcome barriers to adoption.

This innovation is novel in that it actively involves patients in developing a set of tools that will effectively engage them in the 3-step fall prevention process. We were unable to find published examples of institutions that have developed and implemented tools for this purpose. We will start with the provider toolkit that we developed based our previous research[1, 23, 24] and a simple paper tool prototype that is based on a program we saw during a visit to Cathay Hospital in Taiwan where family caregivers are routinely involved in completing a fall risk assessment and in helping the patient to execute the plan during a hospitalization (the fall rate in this hospital is <.5 falls per 1000 patient days on medical units in contrast to US hospitals where fall rates are typically 3-4 falls per 1000 patient days on medical units). Patients, family caregivers, and other key stakeholders will collaborate first in helping us to fully understand the problem of fall prevention from their perspectives and then in a participatory design process to design and develop a fall prevention toolkit. Our team has successfully used these methods in previous studies[25, 28, 29]. Through our collaboration with Montefiore Medical Center in Bronx, NY, we will target patients from different levels of health literacy and ethnic/racial background for participation. The toolkit will adhere to the national standards on *Culturally and Linguistically Appropriate Services (CLAS)*[30] and in so doing, bridge the communication and health literacy gap between professional, paraprofessional and consumer members of the healthcare team.

RESEARCH DESIGN

Expertise of the Research Team

	Fall Prevention	Patient Safety	Patient Engagement	Health Services Research	Informatics	Measurement and Evaluation
Dykes	X	X	X		X	X
Bates	X	X		X	X	X
Adelman	X	X			X	X
Fagan			X			
Lipsitz	X	X		X		X
Benneyan		X				X
Neri		X	X		X	

The research team has broad expertise and has conducted studies in the areas of fall prevention, patient safety, patient engagement, informatics, rapid prototyping, user-centered design, health services research, measurement and evaluation.

Design overview: The project is exploratory and divided into five phases: **1) Problem analysis** using workflow observations, individual and group interviews; **2) Design** using knowledge gained in phase 1 to design a patient-centered fall prevention toolkit; **3) Development** using participatory design, rapid prototyping, and computer modeling and simulation methods to construct the patient-centered fall prevention toolkit; **4) Implementation** of the toolkit on patient care units; and **5) Evaluation** of the toolkit incorporating metrics to address each component of the RE-AIM framework.

The project will be conducted on patient care units in two hospitals using a cluster randomization design with hierarchical modeling (See **Figure 2**) at BWH and a pre/post pilot at Montefiore Medical Center.

R	O_1	$X_{Development}$	X_{FPTK}	O_2	O_3	O_4
R	O_1			O_2	O_3	O_4

Figure 2: Research Design

Key:
R = Randomization
$X_{Development}$ = System Development
X_{FPTK} = Fall Prevention Toolkit
O_1 = Baseline Observation
O_{2-4} = Unannounced Visits

While patients who have been hospitalized in different types of adult units at BWH and Montefiore hospitals will be involved in defining the problem and iteratively designing and developing the intervention, initial testing in year 1 will occur on two oncology units at BWH. These units were selected because our team is currently implementing an electronic bedside communication center[29] on these units that patients can use to access the fall prevention toolkit. Patients with cancer are at increased risk for falls and fall-related injury[31]. The toolkit prototype will also be tested on one medical unit at Montefiore in year 1. In year 2, the fall prevention toolkit will be integrated with the Epic Medical Record (EMR) system at both hospitals and then rolled out to additional oncology and medical units at BWH and one additional medical unit at Montefiore Medical Center in years 2-3. In years 3-4 the toolkit will be rigorously evaluated.

Patient care units will be randomized to receive the FPTK intervention or to serve as a usual care unit. Within the 12 oncology units at BWH, 6 units will be randomized to FPTK intervention and 6 units to usual care. Similarly, within the 8 medical units at BWH, 4 units will be randomized to FPTK intervention and 4 units to usual care. Patients on the usual care unit will receive usual care as it relates to fall prevention. The intervention will integrate knowledge gained from workflow observations and interviews of stakeholders, practice guidelines, the fall prevention literature, and lessons learned from our previous work[1, 24, 25, 28, 29, 32, 33]. Outcome measures are driven by the RE-AIM components to ensure that implementation and dissemination are addressed through all phases of the project.

Phase 1: Problem Analysis Individual and group interviews and work flow analyses will be used to learn about the needs and preferences of patients and providers and other social-technical factors that relate to fall prevention.

<u>*Focus groups and feedback sessions:*</u> We will conduct individual and focus style group interviews throughout the study. The purpose of the interviews in phase I will be to learn current state related to the 3-step fall prevention process from the perspectives of patients/family caregivers, professional and paraprofessional providers. We will follow basic content analysis methods [39] to interpret descriptive data obtained from individual and focus group interviews. In addition, we will conduct focus style group interview sessions with 5 healthcare providers and 5 patient representatives. Patient representatives will be identified through the Patient and Family Advisory Councils at each hospital. Focus style group discussions will be recorded, transcribed, and evaluated as described above will be conducted using a semi-structured interview guide with approximately 4-5 specific guiding topics. We will focus on understanding the barriers and facilitators of fall risk assessment,

Table 1. Fall Prevention Protocol on Usual care and Intervention Units

		Usual Care Units	Intervention Units (FTTK)
1.	Fall Risk Assessment and Planning:<br>▪ Admission<br>▪ Weekly<br>▪ Change in status	Nurse completes Morse Fall Scale (MFS)[29] using existing paper or electronic forms. Follow unit/facility protocol for care planning.	Patient engages with toolkit and collaborates with nurse to complete Morse fall risk assessment, to select personalized, evidence based interventions, and to execute the plan using the toolkit
2.	Bedside Alert to all Stakeholders	Generic "High risk for falls" sign above bed for patients scoring >45 on MFS.	Personalized fall prevention plan is available at bedside on admission; updated with change in status.
3.	Patient Education (usual care and intervention materials available in English/Spanish)	Educate patient/family members providing booklets or other generic fall prevention handouts as needed.	Patient education is streamlined; patient/family members are encourages to continuously engage in the in the 3-step fall prevention process (risk assessment, plan generation and execution) and access and discuss educational material. Additional handouts and generic fall prevention materials are not needed.
4.	Documentation of the Fall Prevention Plan	Fall prevention plan documented in medical record.	Patient's personalized fall prevention plan documented in medical record.

developing a personalized plan, and executing the plan with regard to engaging patients and family caregivers in their care plan. The outcome of phase 1 is a set of requirements for a toolkit to support the 3-step fall prevention process that meets patient/family member expectations and is conducive to existing interdisciplinary workflows. The recommendations will be used to build the patient centered fall prevention toolkit in phase 2.

Focus groups and feedback sessions: In phase 1 and at the conclusion of the intervention period, we will conduct a large multidisciplinary session with patient collaborators/advocates, healthcare providers, and key stakeholders (approximately 20 individuals) in order to better understand barriers and facilitators of patient participation in the fall prevention process (phase 1) and implementation success and effective use of the patient centered fall prevention toolkit going forward (phase 3). We will also use this session to address patient and provider concerns that may arise from interacting via the individual components of the toolkit. The knowledge and feedback gained from this session will be used to inform how to implement these types of interventions in the future.

Workflow analysis: Workflow analyses will be completed to validate interview findings and to explore opportunities for use of electronic tools to improve communication and fall prevention practices on patient care units. Using methods applied in our previous work[28, 34-36] we will observe patient and care team interaction related to the 3-step fall prevention process. Pamela Neri, a human factors expert at Partners, will conduct a series of direct observations of patients and providers during phase 1. The goals of these observations will be to 1) identify current workflow patterns and 2) consider how they might be impacted by the intervention. This information will be used to inform the configuration of the intervention and to anticipate needs for training.

Phase 2: Design. Our team's expertise in medicine, nursing, patient advocacy, engineering, informatics, and usability will be leveraged to ensure that differing perspectives are captured in the design phase. Findings from phase 1 will be used to identify requirements for a patient-centered fall prevention toolkit that addresses stakeholder goals and the tasks necessary to promote engagement in the 3-step fall prevention process. We will define the content, display, and workflow integration strategies most likely to address requirements and overcome barriers identified in phase 1. Common themes will be prioritized, mapped to the 3-step fall prevention process and then used to inform the toolkit prototype. The toolkit components will adhere with national standards on *Cultural and Linguistic Competency*[28] to bridge the communication and health literacy gaps between patient/family caregiver and professional team members. The outcomes of each step will be summarized and used to formulate a report where problems are identified and recommendations prioritized. In high level design, we will take advantage of brainstorming sessions to generate ideas with impact. An initial mockup will be developed and refined by our research team. The prototype will be further refined though focus groups with stakeholders (patients, family, care team members) using develop-test-revise iterations to identify components to be included in the detailed design.

We will then create a detailed design by mapping out the core and interdependent functions of the fall prevention toolkit system along with the specific patient requirements related to participation in each of the 3 steps of the fall prevention process. We will use prototype graphical user interfaces to engage with stakeholders and software simulation to explore the potential impact of the toolkit on workflow. We will leverage the Systems Engineering, Usability and Integration Subcore and the resources in the Methodology

and Resources Core to address needs for standardization, interoperability, redundancy and ultimate integration with the Safety Checklist Tool and MySafeCare toolkits that will be developed simultaneously. Through collaboration with our usability experts, the design process will be informed by end user needs and usability considerations.

Phase 3: Development. We will collaborate with patients/family caregivers to iteratively develop a patient centered fall prevention toolkit. Through our design work, we will identify the fall prevention toolkit components for full scale development. Using methods that we have applied previously[28, 34-36], we will iterative test and evaluate the fall prevention toolkit components with patients, family, and other stakeholders (See **Figure 3**). We will do initial testing using focus groups and interviews until we have a working prototype that stakeholders agree is acceptable. We will then continue testing/developing with hospitalized patients. Throughout the development process, we will continue to use software simulation to explore the potential impact of the toolkit on workflow. Our multidisciplinary team of clinicians, architects, designers, engineers, human factors specialists, and end users will ensure that system requirements and design specifications are fulfilled and that human factors and usability principles are addressed throughout the development process. We will go through an iterative process of prototype refinement and usability testing until a sufficiently mature version of the toolkit has been developed that stakeholders agree will support patient and family engagement in the 3-step fall prevention process without significant usability problems. This version of the toolkit will then be formally evaluated in phase 4.

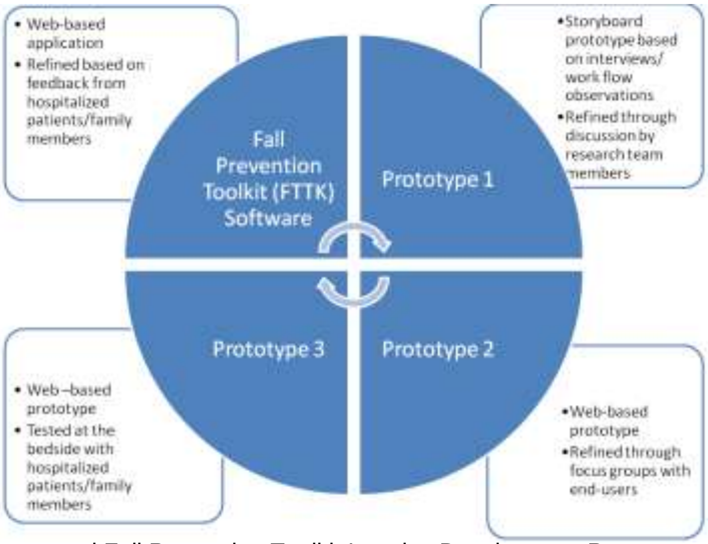

Figure 3: Patient-centered Fall Prevention Toolkit Iterative Development Process

Phase 4: Implementation. We will implement the patient-centered fall prevention toolkit as a single component of a larger integrated suite of tools (Patient-centered Fall Prevention Toolkit, Patient Safety Checklist Tool, and MySafeCare patient reporting system) on clinical units in two hospitals.

During the pilot implementation phase in year 1, we will evaluate the patient-centered fall prevention toolkit in the context of fall prevention practices and the overall workflows on two oncology units at BWH and one medical unit at Montefiore. We will use systems engineering methods and tools including process flow mapping and analysis, work design and simplification, root cause analysis, workload estimation, and general principles from lean and six sigma to evaluate the impact and to refine the toolkit. The toolkit will be accessed through an existing electronic bedside communication center and will allow us to observe the effect of the fall prevention toolkit itself and in the context of the larger integrated suite of tools. For example, what new tasks, procedures and workflow patterns emerge? The pilot implementation will provide an opportunity to enhance the software and to correct any “bugs” that could lead to “workarounds” and impede adoption. We will use predefined RE-AIM metrics (see) **Table 2** to verify whether the fall prevention toolkit is working as intended. For example, are we “reaching” all patients admitted to the pilot units? If not, what are the barriers and what changes can be made before implementing more widely. We will perform a human factors evaluation regarding use of the patient centered fall prevention toolkit by patients and providers. The goals of these observations will be to determine: 1) the facilitators of and barriers to effective use of the toolkit by patients and/or family members; and 2) how communication and collaboration related to the fall prevention process changed from the pre-intervention to intervention period. This information will be used to refine the toolkit and our approach to training patients and providers in the future. The pilot will also provide an opportunity to test the integrated system and identify any socio-technical factors or unintended consequences that may have been unrecognized that could limit effectiveness or create excessive work burden on care team members.

While we estimate that the pilot implementation will last about a month, we will continue until a point of diminishing returns is reached for discovering and correcting overall system vulnerabilities. The Patient-centered Fall Prevention Toolkit will then be implemented. We will use a peer champion model for education and training since this model has worked well in our previous projects[28].

Table 2: Outcome Measures

Measure	Project Phase(s)	Data Source	Method	Analytic Variable(s)
**Reach**				
Use of the toolkit by patients.	Evaluation	Study database<br><br>Patient interviews	Report<br><br>Patient interviews	• Percent and types of patients admitted who use the toolkit.<br>• Description of excluded patients.<br>• Patient perceptions of why they chose or chose not to use the toolkit.
**Effectiveness**				
Positive and negative unanticipated consequences.	Evaluation	Patient interviews	Patient interviews	• Stakeholder perceptions of positive and negative consequences.
Patient activation in the 3-step fall prevention process	Evaluation	Patient Activation[36] Related to Fall Prevention Scale **(Appendix 1)**	Patient interviews	• Proportion of questions with positive responses.
Patient falls<br>Patient falls with injury<br>Effectiveness in older vs. younger patients	Problem Analysis; Evaluation	Event reporting systems	Report	• Proportion of patients with ≥ 1 event (overall and subgroup analysis)
Concordance between patients and providers re: personalized fall prevention plan (PP)	Problem Analysis; Evaluation	Patient, MD, RN interviews **(Appendix 2)**	Interviews	• Proportion of cases with global concordance scores ≥ 6
**Adoption**				
In what percent and types of settings and staff is the innovation adopted? Is the intervention consistent with the values and priorities of the organization and its staff?	Evaluation	Study database<br><br>Patient interviews	Report<br><br>Patient interviews	• Description of use within and across units.<br>• Stakeholder perceptions of toolkit including consistency with workflow, values, priorities.
**Implementation**				
What "bugs" are noted? What "workarounds" develop? How consistently are different parts of the innovation Implemented? What resources were used to support implementation? What barriers and enabling factors were identified related to implementation? How were they addressed? How has communication and collaboration related to the fall prevention process changed from the pre-intervention to intervention period? Are unintended consequences to patient safety or workflows noted?	Implementation	Workflow observations<br><br>Patient, MD, RN interviews		• Description of system performance.<br>• Description of training and associated resources.<br>• Stakeholder perceptions of barriers and enabling factors.<br>• Description of the impact of the system on communication and collaboration related to the 3-step fall prevention process.<br>• Description of workflow changes, unintended consequences.
**Maintenance**				
How well are the innovation components and their effects maintained?<br>What strategies were used to sustain the intervention over time?	Evaluation	Workflow observations<br>Patient Activation[36] Related to Fall Prevention Scale **(Appendix 1)**<br>Patient, MD, RN interviews	Observations<br>Interviews<br>Survey	• Description of system performance over time.<br>• Description of emerging workflow changes, unintended consequences.<br>• Description of the long-term impact on patient activation, care plan concordance, patient falls and related injuries.

Phase 5: Evaluation. After the Patient-centered Fall Prevention Toolkit has undergone implementation testing and further improvement, it will be rolled out to all of the additional oncology and medical units at BWH and 1 additional medical unit at Montefiore where its effectiveness can be more rigorously evaluated. Outcome measures related to each component of the RE-AIM Framework will be tracked (see **Table 2**). In addition to the quantitative measures, we will also conduct focus style group interviews using methods described in Phase 1 to identify patient and care team perceptions of the facilitators and barriers to use of the patient-centered toolkit and recommendations for improvement.

Research Questions: Does use of a patient-centered fall prevention toolkit by patients and family 1) improve patient activation in the 3-step fall prevention process, 2) improve concordance in understanding of the fall prevention plan by patients and providers and 3) lead to fewer falls and injurious falls?

Screening and eligibility: Due to the complexity of acute care workflows, the ethical implications of identifying risk and not intervening, and the quality improvement nature of the intervention, all patients on participating units will be included in the sample. While all patients/family caregivers will be offered the opportunity to use the toolkit intervention to participate in the fall prevention process during the study period, we recognize that patients/family members may choose to participate or not participate or to carry out or not carry out actions to prevent falls. In phase 5 patient interviews, we will explore why some patient chose not to use the toolkit or engage in the 3-step fall prevention process.

Analytic Methods

A cluster RCT design with hierarchical modeling will be used at BWH and a Pre/Post pilot at Montefiore Medical Center. Patient falls and falls with injury will be obtained from each hospital's event reporting system. Use of the toolkit components will be tracked via unannounced site visits. The effect of the intervention on patient falls (dichotomized as fall/no fall) and falls with injury (dichotomized as falls with injury/no injury), will be analyzed using logistic regression estimated via generalized estimating equations (GEE) to account for clustering within unit. The global concordance with fall prevention plan score will be dichotomized (0 to 5 vs. 6 to 12). Logistic regression via GEE to account for clustering within unit will also be used for the latter two outcomes. The additional RE-AIM evaluation measures (see **Table 2**) will be presented descriptively.

General:

We will test the following hypotheses:

(a) Patients/family caregivers who have access to the patient-centered fall prevention toolkit will be more engaged/activated in the 3-step fall prevention process than patients/family caregivers in the usual care arm (Patient Activation Related to Fall Prevention Scale, **Appendix 1**).

(b) Patients/family caregivers and professional providers who have access to the patient-centered fall prevention toolkit will have greater concordance with the personalized fall prevention plan than patients/family caregivers and professional providers in the usual care arm (Concordance in Understanding of Fall Prevention Plan Interview Instrument, **Appendix 2**).

(c) Use of the patient centered fall prevention toolkit will be associated with a decrease in the incidence of patient falls.

(d) Use of the patient centered fall prevention toolkit will be associated with a decrease in the incidence of patient falls with injury.

Statistical techniques: All of the quantitative outcomes are dichotomous. To estimate the treatment effect, we will apply GEE to estimate the logistic regression model for the binary response for each patient in the project. The main predictor is the patient centered fall prevention toolkit effect, which will be tested by comparing the intervention to no intervention (usual care). The model will also include an effect for type of unit (oncology or medical) since units are randomized within the two types.

Outcomes analyses and reporting: Patient, family caregiver and provider characteristics will be presented descriptively using means with standard deviations, medians with inter-quartile ranges, and proportions with 95% confidence intervals as appropriate. Each outcome will be reported as crude and adjusted/clustered effects with 95% confidence intervals and compared between control and intervention arms using GEE. All analyses will be on an intention-to-treat basis.

Focus group analyses and reporting: As done in our previous studies[20,21], the tape-recorded focus group discussions will be held in a private conference room on the units and individual interviews at the patient's bedside. A discussion guide will be used, but the approach will be individualized to guide participants to help us understand what they do and perceptions of barriers and facilitators to the 3-step fall prevention process. See **Appendix 3** for sample interview guide for patients and family members and **Appendix 4** for sample interview guide for interdisciplinary team members and paraprofessionals. Notes will be taken to guide specific follow-up questions. Probes, such as "Tell me some more about…, Help me understand…," will be used. Research team members will take notes during the discussion and keep reflective notes during the qualitative phase. The group interviews will be transcribed verbatim, reviewed/corrected for transcription accuracy and

removal/masking of any identifying characteristics of patients or team members, and converted into NVivo for coding and support of analysis. We will use two-person consensus for the analysis. First we will open code text to capture meanings in the data, compare codes with each other and across units, and perform selective coding to identify core categories. We will use a process of debriefing among researchers, engagement with the raw data and codes, and employ field and reflective notes to assure reliability and validity. We will link core categories in order to discover new perspectives from the accounts of participants' experiences[42] and for developing elements of the patient-centered fall prevention toolkit.

Study population: We will conduct our study in 20 units in BWH (12 oncology and 8 medical) and 2 units at Montefiore Medical Center. Due to the quality improvement nature of the intervention, any patient admitted to these units will be eligible for enrollment during the intervention period. Patients admitted to the acute care units typically have a variety of underlying chronic medical conditions[33] and are typically admitted for evaluation and treatment of an acute illness and/or exacerbation of a chronic illness. These patients typically include several vulnerable populations, including the elderly (BWH and Montefiore) and the underserved (Montiefiore). Montefiore serves an ethnically diverse population in Bronx, NY. According to the 2010 Census, over 30% of the population is non-Hispanic Black or African American and 54% is of Hispanic, Latino, or Spanish origin (any race)[37].

Sample size: All patients admitted to the intervention units will be participants in the project. Based on 2013 discharge data, we expect to enroll 20,694 patients at BWH and 4154 patients at Montefiore Medical Center.

Patient activation and fall prevention plan concordance: These outcomes are collected by questionnaire and thus will be collected in a random subsample of patients. A sample size of 63 patients in each unit for a total of 628 in each of the intervention and control arms will give us 80% power to detect a 10% absolute increase in the proportion of questions in each domain with positive responses (i.e., from 50% to 60%), with a Type I error rate of 0.05. Again, a generalized estimating equations z-test[41] will be used to account for clustering of patients within unit (conservatively assuming the intracluster correlation (ICC) for this outcome among patients within the same unit is 0.01).

The following outcomes will be tested at a Type I error rate of 5% with 80% power.

1. *Patient falls:* Based on 2012 data from all participating facilities, the estimated fall rate was approximately 4.2 falls per 1000 patient days (and an average length of stay of 5 days in medical units and 9 days in oncology units), and we expect the effect size to be a 22% decrease in the fall rate with application of the intervention to 3.27 falls per 1000 patient days. As seen in our previous study[3], we assume the ICC for patients in the same hospital unit is approximately 0.0001. . Using a generalized estimating equations z-test[41] to account for clustering of patients within within unit, an average of at least 1050 patients in each of the __20 units are needed to detect the projected 22% drop in the falls rate for the intervention units versus control units with a two-sided type 1 error rate of alpha=.05 and with 80% power. In particular, 21000 total patients (10,500 in each arm) will give 80% power to detect the expected 22% decrease in the patient fall rate. Based on 2012 data, in which each unit had on average 630 patients for a 12 month period (approximately 1100 for a 21 month period), we will have adequate accrual in these units in a 21 month period to detect the projected decrease in the fall rate.
2. *Patient falls with injury:* Based on 2012 data from all participating facilities, the estimated fall with injury rate was approximately 1.0 falls with injury per 1000 patient days. With __1050_patients per unit for the 21 month period expected for this study, we have 80% power (with alpha=.05) to detect a 43% decrease in the fall with injury rate (to 0.57 falls with injury per 1000 patient days) with application of the intervention. Again, we use a generalized estimating equations z-test[41] to account for clustering of patients within unit assuming the intra-class correlation coefficient for patients in the same hospital unit is approximately 0.0001. Because this sample size will provide 80% power to detect a large (43%) decrease in the patient fall with injury rate we will track falls with injury as a secondary outcome measure.
3. *Patient falls, Montefiore Medical Center pilot:* Based on 2013 data, in the two units studied at Montefiore, we expect 7277 total patients in 21 months pre-period and 7277 patients in the 21 month post-period. In the pre-period, we expect the fall rate to be approximately 4.2 falls per 1000 patient days (and an average length of stay to be 6.8 days). With 7277 total patients in each of the pre and post periods, we have 80% power (with alpha=.05) to detect a 29% decrease in the fall with rate (to 3.02 falls per 1000 patient days) from pre to post. Here, with only two units, we use a logistic regression z-test for the pre-post effect with a fixed effect for unit.

Cores Accessed

To complete this project we will access the Methodology and Data Resources Subcore and the Dissemination and Translation Subcore. A broad range of expertise is needed to complete the study aims. The Systems Engineering, Informatics, Usability and Integration Core expertise, and qualitative methods and tools are needed to complete the in-depth problem analysis, design, and development work. Clinical, informatics, performance improvement, patient safety, and usability expertise is needed to support successful implementation. The mixed methods design and complex evaluation plan requires collaboration with methodologists with expertise in epidemiology, biostatistics, qualitative, and health services research. In addition, the Dissemination Core will assist our team with identifying opportunities for collaboration; provide assistance with disseminating key findings, and translating them into practice and policy recommendations.

Timeline

**Table 4**: Patient-centered Fall Prevention Toolkit (FPTK) Timeline	**Year 1**				**Year 2**				**Year 3**				**Year 4**			
Research Projects	Q1	Q2	Q3	Q4	Q1	Q2	Q3	Q4	Q1	Q2	Q3	Q4	Q1	Q2	Q3	Q4
**Develop Patient-centered FPTK**																
Development and pilot-testing	X	X	X	X												
**System Integration**																
EMR/Safety Reporting System					X	X										
**Implementation/Evaluation**																
Training						X	X									
Rollout							X	X								
Data collection	X	X	X	X					X	X	X	X	X	X	X	
Data analysis	X	X	X	X												X
Dissemination			X	X	X	X	X	X	X	X	X	X	X	X	X	X

HUMAN SUBJECTS RESEARCH

Members of the research team, who have all been certified in the protection of human subjects, will use methodologies to assure confidentiality of all data. Every effort will be made to protect the confidentiality of all human subjects by utilizing de-identified institutional databases, removal of all protected health information from medical record data forms, and fall incident reports. Human subjects assurances will be applied for at Partner's HealthCare, the oversight at BWH, and the Institutional Review Board at Montefiore Medical Center.

Protection of Human Subjects

This project will be conducted on adult, non-critical care patient care units at 2 acute care hospitals. All patient data from both the usual care and intervention units will be downloaded from institutional databases where all data is stored with a code. Patient data via log files and other data mining processes that are in place at the individual sites will be used. Data will be stored with no identifiers.

Data from the incident reporting systems as well as audits to evaluate the degree to which the toolkit is used will be stripped of all identifying information and replaced with a randomly assigned code. The key to linking this code with identity of subjects will be electronically maintained in a password-protected network drive database. All stored data will be in a locked file cabinet and/or a password-protected database file specifically designed for this study.

Due to the complexity of acute care workflows, the ethical implications of identifying risk and not intervening and the quality improvement nature of the intervention, all patients on participating units will be included in the sample. Therefore, the study population will be representative of the population that stands to potentially benefit from this research.

Adequacy of protection against risks. All investigators and research study personnel who interact with the human subject data are certified in the protection of research subjects. Potential subjects will be identified by the research team from daily census on the study patient care units at each institution. The data from this study will be collected primarily through institutional databases, fall incident reports, and medical record chart audits. The data will be accessed by study personnel for data entry and analysis. Data will be monitored by the Principal Investigator and by the Co-investigator responsible for the database on a regular basis. Monitoring will include assessment of the integrity of the study procedures, data completion and quality.

Potential benefits of the proposed research to the subjects and others. There are no known benefits to participation in this study, but the information from this study may identify appropriate interventions for engaging patients and family in the prevention of falls and other adverse events in acute care hospitals.

Importance of the knowledge to be gained. The information gained from this proposal will provide data to prevent patient injury associated with the delivery of health care in acute care short stay hospitals and may inform public policy related to inclusion of patient falls as a preventable hospital acquired condition.

Inclusion of Women and Minorities. All male and female patients and any ethnic or minority groups that are patients on the study units are considered potential subjects for this study. The area from which the BWH draws patients includes sections around the city of Boston, which has diverse ethnic groups of Hispanic, Asian and African-Americans. Montefiore draws patients from ethnically diverse sections of Bronx and Westchester County in New York State. See Planned Enrollment Tables.

Inclusion of Children. Although children comprise a population cared for in acute short stay hospital, they are not included in this study because their fall risks are different from adults. National Database of Nurse Sensitive Quality Indicators (NDNQI) exempts reporting of children in this national database.

Data Safety and Monitoring Plan

Based on an assessment of risk, this study represents minimal risk in that the probability and magnitude of harm or discomfort anticipated in the research are not greater in and of themselves than those ordinarily encountered in daily life or during the performance of routine physical or psychological examinations or tests. [45 CFR 46.102(i) and 21 CFR 56.102(i)].

The data safety and monitoring plan for this study will include safety and adverse event reporting to the NIH and the Institutional Review Boards. An adverse event is defined as any unfavorable and unintended sign, symptom or disease associated with the study treatment that occurs during the course of the study. Adverse events scores are in **Table 3**. We do not anticipate any moderate, severe, life-threatening or fatal adverse events caused by the proposed study. The Principal Investigator, in consultation with the team, is responsible for evaluating affects the risk/benefit ratio of the study and whether modifications to the protocol and the consent form are required.

**Table 3. Adverse Event Scores**	
0	No adverse event
1	Mild – did not require treatment
2	Moderate – resolved with treatment
3	Severe – results inability to carry out normal activities and requires medical attention
4	Life-threatening – results in immediate risk of death
5	Fatal

The Principal Investigator (PI) of this project, Dr. Patricia Dykes, has primary responsibility for the overall conduct of this study and for the safety of participating human subjects. The PI will ensure that the informed consent process is conducted appropriately and that informed consent is obtained prior to proceeding with any study procedures; that only eligible subjects, per protocol eligibility criteria, are enrolled in the study; data are collected and analyzed per protocol; that the privacy and confidentiality of the human subject is maintained; procedures are implemented to ensure that the project is consistently monitored for possible adverse events; adverse events are reviewed promptly and reported as required to the Institutional Review Boards. While implementation of the data safety and monitoring plan may be delegated to members of the research team, the PI maintains ultimate responsibility for the study and for the safety of the human subjects.

The Co-investigator, Dr. Adelman, will participate in the monitoring of safety of the human subjects together with the PI. On a semi-annual basis, Dr. Dykes will prepare a written report on the progress of the study including data regarding: enrollments, comparisons with target to actual enrollment, overall status of the study participants, information on race and ethnicity, age, gender, attrition and any adverse events. The PI and the research team will meet to discuss these reports on a semi-annual basis. During the review of these reports, the PI and research team will determine whether additional effort is required to foster the progress of the study, whether adverse events have occurred, whether adverse events were dealt with appropriately and whether adverse events were correctly and immediately reported to the Institutional Review Boards and the NIH. The PI and research team will determine whether the study should continue, be terminated, or be modified based on observed beneficial or adverse effects.

The PI is responsible for reporting adverse events to all members of the research team and to the Institutional Review Boards and NIH. Mild expected and unexpected adverse events will be summarized in the progress report at continuing review. Moderate and serious unexpected adverse events will be reviewed and reported by

the principal investigator and reported to the IRB according to Partners and Montefiore AE Reporting policies. All of the research team members will be trained in recognition, response and recording of adverse events to ensure the safety of the human subject; and to report these events to the PI in a timely manner to ensure compliance with institutional policies in human subject protection.

As this is a no more than minimal risk study, a data safety and monitoring board is not required.

Vertebrate Animals

None

Select Agent Research

NA

Multiple PD/PI Leadership Plan

NA

Consortium/Contractual Arrangements

See Montefiore Subcontract

Letters of Support (e.g., Consultants)

See letters of support within Administrative Core.

Resource Sharing Plan(s)

The Methodology, Data and Resources Core will be responsible for establishing and maintaining a central repository for primary data collected by the program, as well as for the secondary data derived from external sources. This will ensure that the highest standards of data security and subject confidentiality are maintained. By maintaining the data centrally within Partners secured system, we will reduce data security costs. The Program Director will maintain responsibility of providing access to the shared file area for investigators participating in the BWH PSLL.

The Center will make available for widespread distribution practice intervention and implementation methods, lessons learned, study results and toolkits generated from these projects. We will design our materials and toolkits in such a manner that they will be generalizable and easily customizable for adoption and immediate use in other healthcare settings. Results of research findings will be presented at relevant scientific meetings/conferences and submitted for publication to appropriate peer-reviewed scientific journals. We will also develop a website and provide links to published reports for easier access to interested HIT and healthcare policy researchers.

The Translational/Dissemination Core will be responsible for disseminating study results to the larger healthcare community through collaborations with entities such as the Institute for Healthcare Improvement, the Massachusetts Coalition for the Prevention of Medical Errors, the American Medical Informatics Association, the Society of Hospital Medicine and others (See Dissemination Plan under the Translation/Dissemination Core description).

LITERATURE CITED

1. Dykes, P.C., et al., *Fall prevention in acute care hospitals: a randomized trial.* JAMA, 2010. **304**(17): p. 1912-8.
2. Morse, J., R. Morse, and S. Tylko, *Development of a scale to identify the fall-prone patient. .* Canadian Journal on Ageing, 1989. **8**(4): p. 366-377.
3. Morse, J.M., *Preventing Patient Falls: Establishing a Fall Intervention Program* Vol. 2. 2008, New York: Springer. 192.
4. Glasgow, R.E., et al., *The RE-AIM framework for evaluating interventions: what can it tell us about approaches to chronic illness management?* Patient Educ Couns, 2001. **44**(2): p. 119-27.
5. Glasgow, R.E., T.M. Vogt, and S.M. Boles, *Evaluating the public health impact of health promotion interventions: the RE-AIM framework.* Am J Public Health, 1999. **89**(9): p. 1322-7.
6. NQF. *Serious Reportable Events in Healthcare: 2005-2006 Update* 2008 [cited 2011 April 4]; Available from: http://www.qualityforum.org/projects/completed/sre/.
7. CDC, *Fatalities and Injuries from Falls Among Older Adults --- United States, 1993--2003 and 2001--2005*, in *Morbidity and Mortality Weekly Report*2006, Centers for Disease Control and Prevention: Atlanta. p. 1221-1224.
8. CDC. *Web-based Injury Statistics Query and Reporting System*. 2010 [cited 2010 April 17]; Available from: http://www.cdc.gov/injury/wisqars/index.html.
9. Tinetti, M.E., et al., *Fear of falling and fall-related efficacy in relationship to functioning among community-living elders.* J Gerontol, 1994. **49**(3): p. M140-7.

10. Tinetti, M.E. and L. Powell, *Fear of falling and low self-efficacy: a case of dependence in elderly persons.* J Gerontol, 1993. **48 Spec No**: p. 35-8.
11. CDC. *Costs of Falls Among Older Adults*. 2011; Available from: http://www.cdc.gov/HomeandRecreationalSafety/Falls/fallcost.html.
12. CMS. *Strengthening Medicare: Better Health, Better Care, Lower Costs: Efforts Will Save Nearly $120 Billion for Medicare Over Five Years*. 2011 [cited 2011 December 28]; Available from: http://www.cms.gov/apps/files/medicare-savings-report.pdf.
13. NQF. *National Quality Forum (NQF) Endorsed Nursing-Sensitive Care Performance Measures* 2009 [cited 2010 April 11]; Available from: http://www.jointcommission.org/national_quality_forum_nqf_endorsed_nursing-sensitive_care_performance_measures/
14. Tinetti, M.E., D. Richman, and L. Powell, *Falls efficacy as a measure of fear of falling.* J Gerontol, 1990. **45**(6): p. P239-43.
15. CMS. *Hospital-Acquired Conditions (Present on Admission Indicator)*. 2009; Available from: http://www.cms.hhs.gov/HospitalAcqCond/01_Overview.asp#TopOfPage.
16. Healthypeople.gov. *http://www.healthypeople.gov/2020/topicsobjectives2020/overview.aspx?topicid=24*. 2011 [cited 2011 January 25]; Available from: http://www.healthypeople.gov/2020/topicsobjectives2020/overview.aspx?topicid=24.
17. Coussement, J., et al., *Interventions for preventing falls in acute- and chronic-care hospitals: a systematic review and meta-analysis.* J Am Geriatr Soc, 2008. **56**(1): p. 29-36.
18. Cumming, R.G., et al., *Cluster randomised trial of a targeted multifactorial intervention to prevent falls among older people in hospital.* Bmj, 2008. **336**(7647): p. 758-60.
19. Cusimano, M.D., J. Kwok, and K. Spadafora, *Effectiveness of multifaceted fall-prevention programs for the elderly in residential care.* Inj Prev, 2008. **14**(2): p. 113-22.
20. Cameron, I.D., et al., *Interventions for preventing falls in older people in nursing care facilities and hospitals.* Cochrane Database Syst Rev, 2010(1): p. CD005465.
21. Oliver, D., et al., *Strategies to prevent falls and fractures in hospitals and care homes and effect of cognitive impairment: systematic review and meta-analyses.* Bmj, 2007. **334**(7584): p. 82.
22. Oliver, D., A. Hopper, and P. Seed, *Do hospital fall prevention programs work? A systematic review.* J Am Geriatr Soc, 2000. **48**(12): p. 1679-89.
23. Carroll, D.L., P.C. Dykes, and A.C. Hurley, *Patients' perspectives of falling while in an acute care hospital and suggestions for prevention.* Appl Nurs Res, 2010. **23**(4): p. 238-41.
24. Dykes, P.C., et al., *Why do patients in acute care hospitals fall? Can falls be prevented?* J Nurs Adm, 2009. **39**(6): p. 299-304.
25. Hurley, A.C., et al., *Fall TIPS: validation of icons to communicate fall risk status and tailored interventions to prevent patient falls.* Stud Health Technol Inform, 2009. **146**: p. 455-9.
26. Dykes, P.C., et al., *A case control study to improve accuracy of an electronic fall prevention toolkit.* AMIA Annu Symp Proc, 2012. **2012**: p. 170-9.
27. Nyberg, L. and Y. Gustafson, *Patient falls in stroke rehabilitation. A challenge to rehabilitation strategies.* Stroke, 1995. **26**(5): p. 838-42.
28. Dykes, P., et al., *Fall TIPS: strategies to promote adoption and use of a fall prevention toolkit.* AMIA Annu Symp Proc, 2009. **2009**: p. 153-7.
29. Dykes, P.C., et al., *Building and testing a patient-centric electronic bedside communication center.* Journal of gerontological nursing, 2013. **39**(1): p. 15-9.
30. N, B., et al., *Literacy and health outcomes*, 2004, Agency for Healthcare Research and Quality: Rockville, MD.
31. Allan-Gibbs, R., *Falls and hospitalized patients with cancer: a review of the literature.* Clin J Oncol Nurs, 2010. **14**(6): p. 784-92.
32. Zuyev, L., et al., *Tailored Prevention of Inpatient Falls: Development and Usability Testing of the Fall TIPS Toolkit.* Comput Inform Nurs, 2010.
33. Zuyev, L., et al., *Tailored prevention of inpatient falls: development and usability testing of the fall TIPS toolkit.* Comput Inform Nurs, 2011. **29**(2): p. 93-100.
34. Dykes, P.C., et al., *The feasibility of digital pen and paper technology for vital sign data capture in acute care settings.* AMIA Annu Symp Proc, 2006: p. 229-33.
35. Dykes, P.C., et al., *A randomized trial of standardized nursing patient assessment using wireless devices.* AMIA Annu Symp Proc, 2007: p. 206-10.
36. Dykes, P.C., et al., *Workflow analysis in primary care: implications for EHR adoption.* AMIA Annu Symp Proc, 2005: p. 944.
37. Borough, U.C. *Bronx borough, Bronx County, New York*. 2010 [cited 2014 January 11]; Available from: http://factfinder2.census.gov/faces/nav/jsf/pages/community_facts.xhtml.
