# Supplementary material for: Evaluation of a Patient-Centered Fall-Prevention Tool Kit to Reduce Falls and Injuries: A Nonrandomized Controlled Trial
Source: JAMA Netw Open. 2020 Nov 17;3(11):e2025889. doi: 10.1001/jamanetworkopen.2020.25889 (PMC7672520; doi:10.1001/jamanetworkopen.2020.25889)
Supplement: Supplement 2. — Data Sharing Statement [file jamanetwopen-e2025889-s002.pdf]

Data Sharing Statement

Dykes. Evaluation of a Patient-Centered Fall-Prevention Tool Kit to Reduce Falls and Injuries. *JAMA Netw Open*. Published November 17, 2020. 10.1001/jamanetworkopen.2020.25889

Data
Data available: No

Additional Information
Explanation for why data not available: Data sharing is not available. The study protocol and statistical analysis plans are available upon request.
